# Enhanced Immune Response to DNA Vaccine Encoding *Bacillus anthracis* PA-D4 Protects Mice against Anthrax Spore Challenge

**DOI:** 10.1371/journal.pone.0139671

**Published:** 2015-10-02

**Authors:** Na Young Kim, Dong Suk Chang, Yeonsu Kim, Chang Hwan Kim, Gyeung Haeng Hur, Jai Myung Yang, Sungho Shin

**Affiliations:** 1 Department of Life Science, Sogang University, Seoul, Republic of Korea; 2 Agency for Defense Development, Daejeon, Republic of Korea; Loyola University Chicago, UNITED STATES

## Abstract

Anthrax has long been considered the most probable bioweapon-induced disease. The protective antigen (PA) of *Bacillus anthracis* plays a crucial role in the pathogenesis of anthrax. In the current study, we evaluated the efficiency of a genetic vaccination with the fourth domain (D4) of PA, which is responsible for initial binding of the anthrax toxin to the cellular receptor. The eukaryotic expression vector was designed with the immunoglobulin M (IgM) signal sequence encoding for PA-D4, which contains codon-optimized genes. The expression and secretion of recombinant protein was confirmed *in vitro* in 293T cells transfected with plasmid and detected by western blotting, confocal microscopy, and enzyme-linked immunosorbent assay (ELISA). The results revealed that PA-D4 protein can be efficiently expressed and secreted at high levels into the culture medium. When plasmid DNA was given intramuscularly to mice, a significant PA-D4-specific antibody response was induced. Importantly, high titers of antibodies were maintained for nearly 1 year. Furthermore, incorporation of the SV40 enhancer in the plasmid DNA resulted in approximately a 15-fold increase in serum antibody levels in comparison with the plasmid without enhancer. The antibodies produced were predominantly the immunoglobulin G2 (IgG2) type, indicating the predominance of the Th1 response. In addition, splenocytes collected from immunized mice produced PA-D4-specific interferon gamma (IFN-γ). The biodistribution study showed that plasmid DNA was detected in most organs and it rapidly cleared from the injection site. Finally, DNA vaccination with electroporation induced a significant increase in immunogenicity and successfully protected the mice against anthrax spore challenge. Our approach to enhancing the immune response contributes to the development of DNA vaccines against anthrax and other biothreats.

## Introduction

Anthrax, a disease caused by the gram-positive, spore-forming, rod-shaped bacterium *Bacillus anthracis*, is highly lethal and an appealing biological weapon owing to its great durability and longevity [1–3]. Since the intentional contamination of mail with *B*. *anthracis* spores in the United States in 2001, there is also increasing concern about its use in terrorist attacks. There are three forms of anthrax characterized by the route of infection, namely, cutaneous, gastrointestinal, and inhalational. The most dangerous form, inhalational anthrax, results from inhalation of spores aerosolized in a particle size small enough to reach the alveoli. The spores are ingested by alveolar macrophages, and surviving spores are then transported to the mediastinal lymph nodes. From there, rapid germination occurs resulting in a fatality rate approaching 100% if left untreated [4,5].

The principal virulence factor of *B*. *anthracis* is a multicomponent toxin secreted by the organism that consists of three separate gene products designated as protective antigen (PA), lethal factor (LF), and edema factor (EF). The pXO1 plasmid is responsible for gene encoding the three toxin components. PA, an 83-kDa protein (PA83), binds to a receptor on the cell surface and subsequently undergoes furin-mediated cleavages to yield a physiologically active 63-kDa form (PA63). The PA63 forms a heptameric complex on the cell surface capable of interacting with either LF or EF, which is subsequently internalized [6,7]. LF is a zinc-dependent metalloprotease, which can digest isoforms of mitogen-activated protein kinases. The resulting disruption of the cellular signaling cascade eventually leads to cell death. EF is a calmodulin-dependent adenylate cyclase that causes degradation of cellular physiology, leading to tissue edema. The LF protein, referred to as LeTx when forming a complex with PA, is considered responsible for the rapid lethality of the anthrax spore inhalation infection [8,9].

It is widely accepted that a major facet of protection against anthrax is an effective humoral immune response against PA [10,11]. Antibodies generated against PA are sufficient for providing protection against the toxin and spore challenge in animal models of anthrax [12–14]. The currently licensed anthrax vaccines for humans consist mainly of PA antigen [15]. However, multiple inoculations are required for a base immunization, followed by yearly boosters to ensure protection. Therefore, they are not ideally suited for the immunization of a large number of individuals, where anthrax is endemic. These vaccines are prepared from filtered culture supernatants of non-encapsulated *B*. *anthracis*. They are expensive to produce and difficult to standardize owing to batch-to-batch variation. The limitations of the presently licensed vaccines have raised widespread interest in developing improved anthrax vaccines.

DNA vaccines deliver genes encoding protein antigens into host cells, enabling antigen production to occur *in vivo* [16]. There are many advantages associated with DNA vaccines when compared to traditional vaccines, which utilize the protein or microorganism itself for immunization. DNA vaccination results in stimulation of both a strong cellular and humoral immune response. In addition, highly clean vaccines can be produced, since DNA vaccines can be designed to produce immunity against a specific target protein. Furthermore, the ability to genetically manipulate DNA is an advantage because vaccines are designed to target protein production in specific cell compartments in order to modulate the specificity of the immune response. The speed with which genetic manipulation may be carried out allows for rapid production of DNA vaccines. A further advantage of DNA vaccines over protein is ease of production and storage [17].

Data from recent studies have demonstrated that immunizations with plasmid DNA encoding the *B*. *anthracis* PA can protect against LeTx challenge in mice or spore challenge in rabbits [18–20]. It has also been suggested that in addition to PA, truncated PA such as PA 63 and domain 4 of PA also play an important role in generating immunity [21,22]. Recently, many attempts have been made to enhance the protective efficiency of DNA vaccines against anthrax, including formulation of PA in adjuvants and use of cationic lipids [19,23]. However, several of these DNA vaccines induced low levels of neutralizing antibodies against anthrax with consequently partial or short-term protection in different animal models.

Therefore, in the present study, to improve the immune response of the anthrax DNA vaccine, we developed and tested DNA vaccines encoding the codon-optimized PA-D4 protein fused with signal peptides and containing the SV40 enhancer. In addition, we also demonstrate the potential of the PA-D4-expressing DNA vaccine administered with electroporation against anthrax.

## Materials and Methods

### Generation of a synthetic codon-optimized PA-D4 gene

The nucleotide sequence encoding *B*. *anthracis* PA-D4 protein (GenBank accession no. AY428556) was codon-optimized with respect to most commonly used codons in human cells by using Genescript OptimumGene^TM^ design platform, which employs a unique algorithm and proprietary codon usage table. A full-length of the PA-D4 gene was chemically synthesized (GeneScript, Piscataway, USA).

### Construction of DNA vaccines

Three different expression plasmids were constructed for the DNA vaccination: (i) We cloned PA-D4 antigens into the mammalian expression plasmid, pEGFP-C1 (Clontech, Mountain View, USA), which contains the gene for the enhanced green fluorescent protein (EGFP). The 419-base pair (bp) codon-optimized PA-D4 genes were inserted into pEGFP-C1 on restriction sites *Eco*RI and *Sal*I to form pEGFP/D4. (ii) PA-D4 was also cloned into the mammalian expression vector pcDNA 3.1 (Invitrogen, Carlsbad, USA), and expression was driven by the CMV promoter. A codon-optimized PA-D4 gene was fused with a nucleotide sequence encoding the immunoglobulin M (IgM) or immunoglobulin kappa (IgK) signal sequence. The pcDNA 3.1 was digested with *Eco*RI and *Xho*I and the PA-D4 gene containing the signal sequence was inserted into the corresponding sites to obtain PA-D4 expression vector IgM-D4 and IgK-D4, respectively. (iii) Plasmids carrying the enhancer from Simian Virus 40 (SV40) were also cloned. A single copy of the 72-bp element from the SV40 enhancer was inserted into the *Bam*III/*Nru*I site located upstream of the IgM-D4 vector. This construct will be referred to as IgM-D4/SV40.

### PA-D4 expression

To confirm the expression of PA-D4, 293T cells were plated at 1×10^6^ cells per well into six-well plates. Cells were transfected with 1 μg of plasmid DNA using the transfection agent Vivagen (Vivagen, Seoul, Korea) according to the manufacturer’s guidelines. After 48 h, cells were harvested and lysed. The cell lysates were separated by 4–20% polyacrylamide gel electrophoresis. Proteins were then transferred from the gel to polyvinylidene difluoride (PVDF) membranes. Membranes were blocked for 1 h in 5% nonfat dry milk in TBST (10 mM Tris-HCl, pH 7.5, 150 mM NaCl, and 0.1% Tween 20) and probed for 1.5 h with primary mouse anti-PA-D4 sera diluted 1:5,000 in TBST/5% nonfat skim milk. Membranes were then washed with TBST 3 times and incubated for 1 h with goat anti-mouse IgG conjugated to horseradish peroxidase (Santa Cruz Biotech, Santa Cruz, USA) at a 1:5,000 dilution in TBST. After additional 4 washes with TBST, the bound antibody was visualized using Western Lighting Plus-ECL (PerkinElmer, Waltham, USA).

We also constructed plasmids that expressed PA-D4 antigens as fusion proteins with enhanced green fluorescent protein as described above. Expression of EGFP was determined using a confocal laser-scanning microscope (LSM 5; Carl Zeiss, Oberkochen, Germany) at 48 h after transfection.

### Capture ELISA of secreted PA-D4

The amount of PA-D4 protein secreted into the cell culture supernatant by cells transfected with plasmids was determined at 48 h post transfection. Briefly, 96-well microtiter plates were coated with anti-PA monoclonal antibody (10 μg/ml) overnight at 4°C. Plates were washed with wash buffer (PBS containing 0.02% Tween 20) and were blocked with 1% skim milk for 1 h before adding samples. Cell culture supernatants were serially diluted in skim milk blocking solution and the plate were incubated at 37°C for 1 h. After washing, mouse anti-PA-D4 antibody (10 μg/ml) was added to the plates and incubated for an additional 1 h at 37°C. Binding was detected by incubation with horseradish peroxidase-conjugated goat anti-mouse secondary antibody diluted in blocking solution for 1 h at 37°C. The plates were then washed 3 times, and the bound antibody was detected colorimetrically using TMB (3,3′,5,5′-tetramethyl benzidine substrate solution) as a substrate, and the A_405_ values of the plates were measured. The amount of secreted PA-D4 of each sample was determined using the standard curve.

### Immunization procedure

Experiments with animals were conducted in compliance with ethical principles in animal experimentation stated in the Sogang University of Animal Experimentation and approved by the Animal Use Ethical Committee at the Sogang University, College of Natural Sciences (approval ID: 201101). DNA delivery in mice was performed either by conventional intramuscular (IM) administration or by IM administration combined with electroporation. Six- to 8-week-old female BALB/c mice were obtained from Samtako (Osan, Korea). Mice were injected intramuscularly in the quadriceps 3 times at 2-week intervals with plasmid DNA dissolved in PBS. As a control, mice were vaccinated control vector (pcDNA 3.1) as above. For electroporation, mice were anesthetized intraperitoneally with Zoletil at a dose of 2 mg/kg before plasmid DNA administration. The skin overlying the quadriceps muscle was shaved and the mice were injected with various amounts of plasmid DNA as described. Two-needle array electrodes (BTX, Hollison, USA) were inserted into the muscle immediately after IM administration of DNA for electroporation. The distance between the electrodes was 5 mm and the array was inserted longitudinally relative to the muscle fibers. *In vivo* electroporation parameters were as follows: 90 V/mm distance between the electrodes, 25-ms pulse length, 3 pulses with reversal of polarity after each pulse, and administered using a BTX ECM-830 electroporator (Hollison, USA). Mice were monitored continually after the procedure until they recovered full mobility, approximately 1 h after the onset of anesthesia. For guinea pig vaccination, 3- to 4-year-old female guinea pigs (Central Lab Animal Inc. Seoul, Korea) were administered 100–500 μg of plasmid DNA into the muscle after shaving and anesthesia. Electroporation was performed using a two-needle electrode array with a 10 mm electrode distance. Three electroporation pulses of 90 V/mm, 25-ms pulse length were given by a BTX ECM-830 electroporator. Sera were collected by retro-orbital bleeding and stored at -20°C until assay.

### Measurement of serum antibody titers

The quantity of antibody present in the serum of vaccinated mice was measured by ELISA using standard procedures. Microtiter plates were coated with *Escherichia coli*-expressed PA-D4 protein (1 μg/ml) in PBS overnight at 4°C. Plates were then washed 3 times in washing buffer (PBS containing 0.02% Tween 20) and blocked for 1 h with blocking buffer (2% skim milk in PBS). Serum samples diluted in blocking buffer were then added and incubated for 1 h, washed 3 times with washing buffer, and the appropriate horseradish peroxidase-conjugated added prior to incubation for 1 h. The bound antibodies were detected colorimetrically. Endpoint titers were defined as the highest dilution of sample with an optical density (OD) reading of greater than 0.1 after subtraction of the absorbance reading of the unvaccinated mice serum samples at the same dilution. For antibody isotyping, a Rapid ELISA Mouse mAb Isotyping Kit (Pierce, Rockford, USA) was used.

### ELISPOT assay

IFN-γ responses specific for PA-D4 were determined using a mouse IFN-γ-specific ELISPOT kit as described by the manufacturer's protocol (Mabtech, Nacka Strand, Sweden). Briefly, multi-screen filtration plates were coated with 15μg/ml anti-mouse IFN-γ antibodies. After overnight incubation at 4°C, the wells were washed and blocked with blocking solution. The spleen cell suspensions from immunized mice were added to the wells at a concentration of 5 × 10^5^ cells per well and re-stimulated for 24 h with antigen (10 μg/ml). After extensive washes with PBS, the plates were incubated with biotinylated anti-mouse IFN-γ antibody for 2 h and with streptavidin-alkaline phosphatase for 1 h at room temperature. Finally, spots were developed using BCIP/NBT-plus as the substrate. The number of IFN-γ-producing cells was determined with the EliSpot Reader System ERL02 (AID, Strassberg, Germany).

### Biodistribution and plasmid quantification

Biodistribution of the DNA vaccine was determined by measuring plasmid copy number (PCN) in diverse tissues by quantitative real-time polymerase chain reaction (qPCR) assay. Isolation of DNA from tissues was performed as described previously [24]. Briefly, BALB/c mice were administered a single dose of 3.5 × 10^13^ copies of IgM-D4/SV40 plasmid DNA. In the control group, mice were immunized with pcDNA 3.1. At various time points following IM administration of plasmid DNA, tissue samples were obtained including the brain, heart, lung, liver, spleen, kidney, muscle, and blood. Subsequently, samples were homogenized using a BioMasher (Nippi, Tokyo, Japan) or a glass homogenizer. The DNA was purified with the DNeasy Blood and Tissue Kit (Qiagen, Valencia, USA).

Quantitative real-time PCR (qPCR) was performed to assess the level of DNA vaccine in tissue samples. PCR was performed using Roche LC480 LightCycler (Roche, Basel, Switzerland) in 96-well plates. The PA-D4 primers for IgM-D4/SV40 detection were 5’ GAGGGACTGCTGCTGAACATTGA 3’ (forward primer) and 5’ GATAAAGGTCTTGCCATCCTGCC 3’ (reverse primer), generating a 156-bp product. Each PCR reaction contained 10 μl of KAPA SYBR FAST qPCR Master Mix Universal (KAPA Biosystems, Boston, USA), 1 μl of each primer at a concentration of 3 pM, 30 ng template DNA, and sterile water totaling 20 μl. After a 5 min preincubation at 95°C, DNA was amplified by 45 cycles of denaturation at 95°C for 10 s, annealing at 50°C for 10 s, and extension at 72°C for 15 s. Melting curves were constructed and agarose gel electrophoresis was performed to confirm the amplification product specificity and size, respectively.

Absolute quantification determines the exact copy number of target gene by relating the threshold cycle (Ct) value to a standard curve [25]. A ten-fold serial dilution series of the IgM-D4/SV40 plasmid, ranging from 1 × 10^2^ to 1 × 10^8^ copies/μl, was used to construct the standard curve. Ct values in each diluted standard were measured in triplicate and were plotted against their initial PCNs. The linearity of each standard curve was assured by the R^2^ (coefficient of determination) of the curves, which is considered as reliable when R^2^ > 0.99. The concentration of IgM-D4/SV40 plasmid was measured by micro-spectrophotometer and the corresponding PCN was calculated using the equation below [26].

$$\text{PCN}=6.02\;\times \;10^{23}\;(\text{copy}/\text{mol})\;\times \;\text{DNA amount}\;(\text{g})\;/\;\text{DNA length}\;(\text{bp})\;\times \;660\;(\text{g}/\text{mol}/\text{bp}).$$

### *In vivo* protection

Two weeks after the final immunization, seven-week-old female A/J mice (Central Lab Animal Inc, Seoul, Korea) were challenged with either 3.5 × 10^3^ spores (20 LD_50_) or 9 × 10^3^ spores (50 LD_50_) of *B*. *anthracis* Sterne 34F_2_ strain. The 50% lethal dose (LD_50_) was calculated following the Reed and Muench method [27]. The control group received with pcDNA 3.1. Spores were diluted to the desired concentration in PBS and injected subcutaneously. The mice were monitored for 14 days post challenge to determine their protected status. Spore-challenged mice were observed daily and were euthanized by CO_2_ asphyxiation when one of following symptoms was detected: bleeding through mouth and nose or hunched posture.

### Statistical analysis

Multiple group comparisons were performed using one-way ANOVA followed by Tukey’s test, using GraphadPad Prism software. Comparisons ELISPOT data were made using Student’s *t*-test. The criterion of significance was *P*<0.05.

## Results

### Expression of PA-D4 *in vitro*

**Fig 1:**
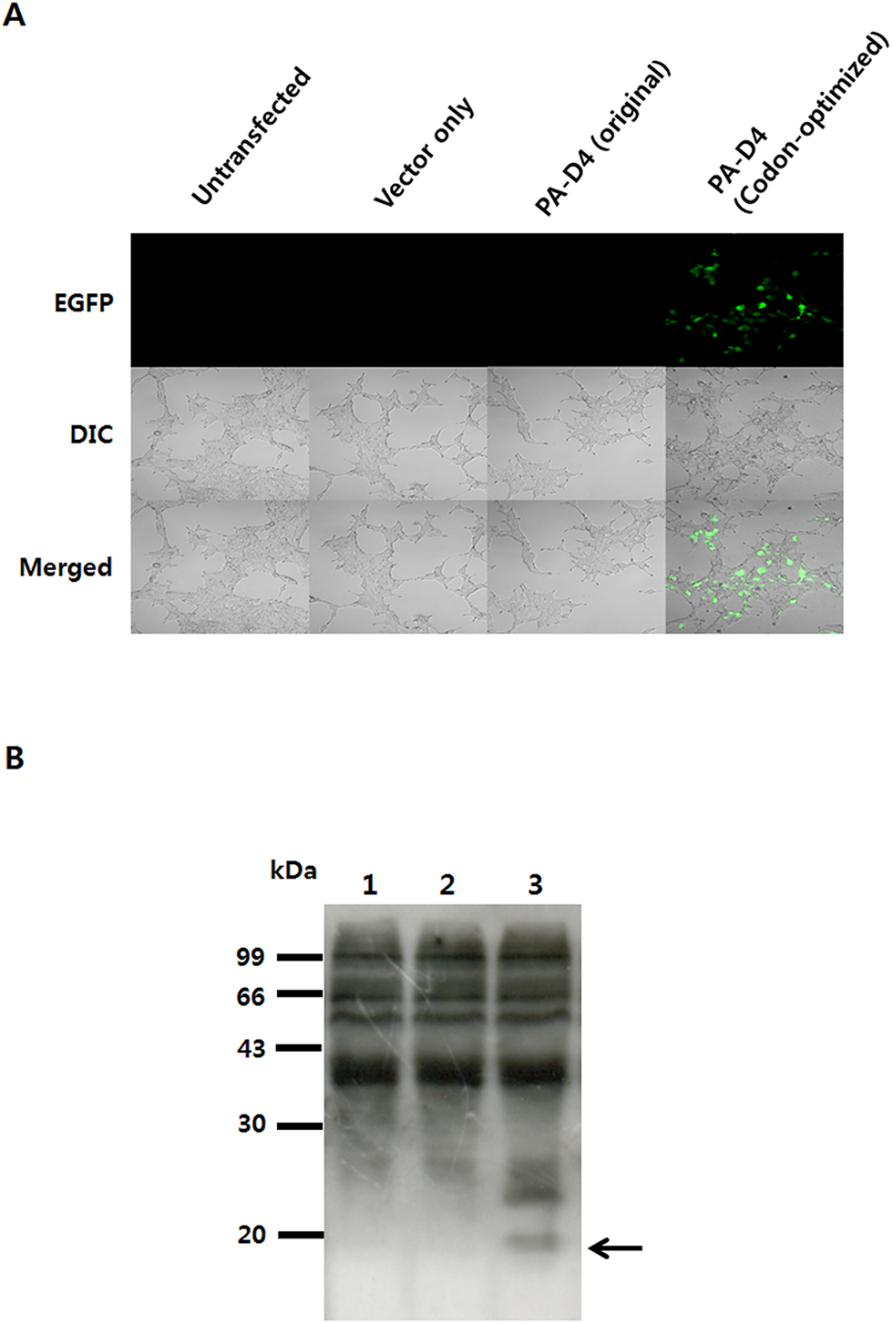
Expression of PA-D4 after *in vitro* transfection. 293T cells were transfected with pEGFP/D4 plasmids. At 48 h after transfection, the cells were harvested and assayed for expression of EGFP fusion PA-D4. (A) Confocal fluorescence microscopy to demonstrate the expression PA-D4 protein. Magnification is 200×. (B) Western blot analysis demonstrating the expression of PA-D4. The proteins were separated in SDS-PAGE using a 4–20% polyacrylamide gel before blotting. Expression of PA-D4 protein was detected with mouse polyclonal antibodies against PA-D4. Lane 1, vector only control cell lysates; lane 2, original type transfected cell lysates; lane 3, codon-optimized transfected cell lysates.

The fourth domain of PA has been shown to mediate binding of PA to its cellular receptor for the anthrax toxin and thus, PA-D4 is a particularly attractive target antigen for the DNA vaccine against anthrax [2]. Therefore, these experiments were carried out using the gene encoding PA-D4, which is a target antigen for the DNA vaccine. A number of studies have found that there is a good correlation between the codon bias of a gene and its level of expression [28,29]. Therefore, we designed a codon-optimized synthetic gene encoding PA-D4, that with most of the rare codons replaced by codons that are most frequently used in human cells. To assess the transfection efficiency of the codon-optimized PA-D4 gene, we decided to construct a eukaryotic expression vector, pEGFP/D4, encoding a PA-D4 with pEGFP-C1 as the backbone. The plasmid vector encoding PA-D4 was transfected into 293T cells. GFP expression was evaluated by fluorescence microscopy 48 h after transfection. As shown in Fig 1A, fluorescence was visualized in cells transfected with codon-optimized PA-D4 but not in original PA-D4 transfected cells. Fluorescence indicates the production of full-length EGFP-D4 fusion protein, since the EGFP component is at the amino terminus.

Further *in vitro* expression was confirmed by western blot analysis. Cells transfected with codon-optimized PA-D4 expressed a protein of approximately 17 kDa, which reacted with anti-PA-D4-specific antibodies. On the contrary, PA-D4 proteins were not detectable in original PA-D4 transfected cells or in vector-only control cells (Fig 1B).

### Construction of plasmids encoding the secretory form of PA-D4 protein

Several reports indicate that the magnitude of the immune response is influenced by the ability of the expression antigen to be secreted [30,31]. Therefore, the codon-optimized PA-D4 open reading frame was fused to the different signal peptides that were shown to promote efficient secretion of heterogeneous proteins [32,33]. To test whether these signal peptides lead to a more efficient secretion, we constructed a plasmid encoding a secreted form of PA-D4, with pcDNA 3.1 as a backbone, containing the cytomegalovirus (CMV) promoter. In plasmid IgM-D4, the PA-D4 encoding sequence is proceeded by the gene encoding the IgM signal sequence, which should direct the PA-D4 gene product to the endoplasmic reticulum (ER) and secretion machinery. The other plasmid, IgK-D4, carries the IgK signal peptide for efficient secretion from the cells.

**Fig 2:**
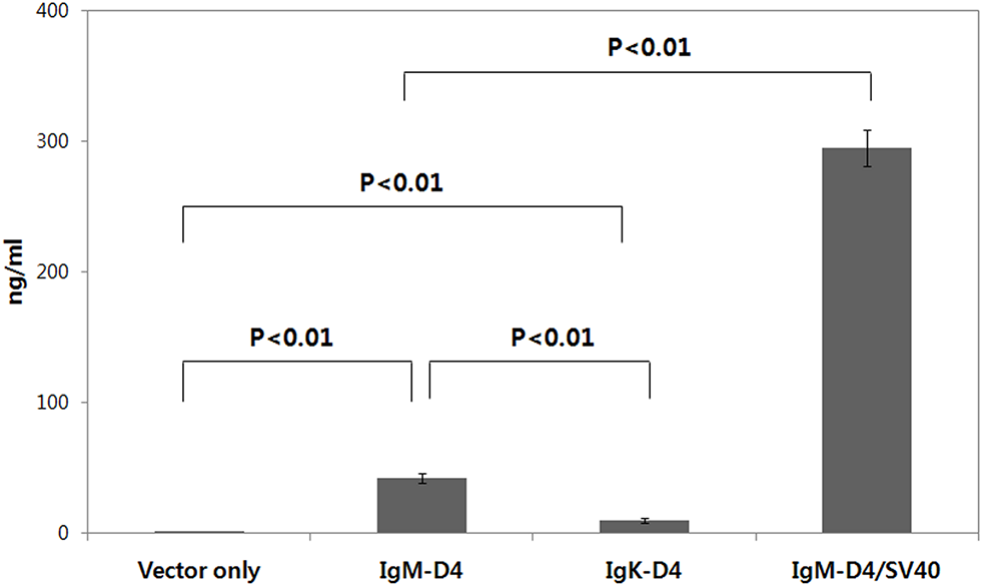
Secreted PA-D4 proteins present in cell culture supernatants were analyzed with capture ELISA. The 293T cells were transfected with IgM-D4, IgK-D4, or IgM-D4/SV40 plasmids. At 48 h after transfection, cell culture supernatants were collected and assayed for secreted PA-D4 proteins with capture ELISA. Values are means ± standard deviations. *P* values were determined using one-way ANOVA followed by Tukey multiple comparison tests.

To determine if PA-D4 could be stably expressed in and secreted from mammalian cells, plasmid IgM-D4 or IgK-D4 was transfected into 293T cells. A comparison of PA-D4 expressed in the culture medium by IgM-D4 and IgK-D4 was made using a capture ELISA. The measurement of expressed PA-D4 by a capture ELISA showed that the level of PA-D4 secreted into the culture supernatant was higher for IgM-D4 than for IgK-D4. As shown in Fig 2, IgM-D4 contained the mouse IgM secretion signal, and 4-fold-more PA-D4 protein was detected in the IgM-D4 cell culture supernatant 48 h post-transfection than in the supernatant from the IgK-D4 cells, which contained a mouse IgK signal.

Several groups have demonstrated that gene expression is increased and made more persistent by the introduction of SV40 enhancer elements into plasmid vectors [34,35]. To test the feasibility of using these DNA elements for gene expression, we cloned 72-bp of SV40 enhancer into an expression vector, IgM-D4. We compared plasmids containing the SV40 (IgM-D4/SV40) with control plasmids (IgM-D4) by transfecting 293T cells. Our results show that incorporation of SV40 enhancer into plasmids leads to strong enhancement of PA-D4 protein secretion (Fig 2).

### Antibody responses after vaccination with plasmid DNA vaccine encoding PA-D4

**Fig 3:**
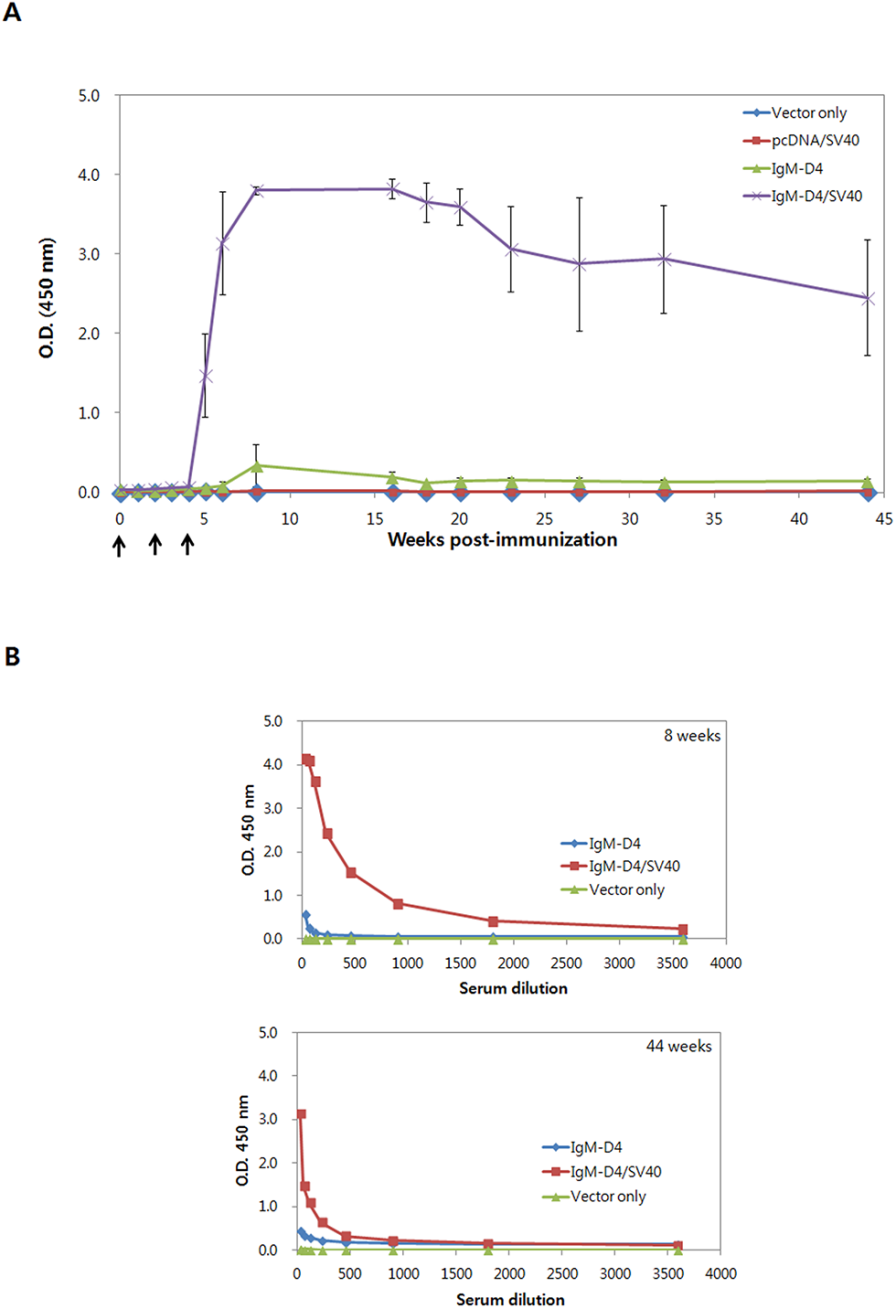
PA-D4-specific antibody responses in mice immunized with IgM-D4 and IgM-D4/SV40 plasmid. (A) Long-term persistence of PA-D4-specific antibodies induced by DNA vaccine. The serum samples from individual mice immunized with plasmid DNA vaccine were collected at various times and the specific anti-PA-D4 antibody responses were analyzed by ELISA using a single dilution (1:100). Mice were immunized three times (week 0, 2, 4) with plasmids as indicated. (B) Induction kinetics of PA-D4 specific antibodies. Serial dilutions of pooled sera from mice were tested in a PA-D4-specific ELISA to determine the kinetics of PA-specific induction. Values are means ± standard deviations.

To assess the immunogenicity responses induced by plasmid DNA immunization, BALB/c mice (*n* = 5–7) were immunized with various DNA constructs by IM injection. Mice were vaccinated 3 times at 2-week intervals for 44 weeks with one of the following plasmids: IgM-D4, IgM-D4/SV40, pcDNA/SV40, or pcDNA 3.1 vector only as a control. Blood was collected at various times following immunization for determination of anti-PA-D4 IgG titers in serum. As shown in Fig 3A, IgG anti-PA-D4 antibodies were induced at 5 weeks after the first immunization with IgM-D4/SV40 and remained elevated for the 44-week duration of the study. The PA-D4-specific IgG were also induced in mice immunized with IgM-D4; however, the titers were lower, by approximately 10 times less than mice vaccinated with IgM-D4/SV40 (Fig 3B). Our results clearly demonstrated that SV40 enhancer is able to significantly increase the rate of antibody production.

### Vaccine dose efficiency

**Fig 4:**
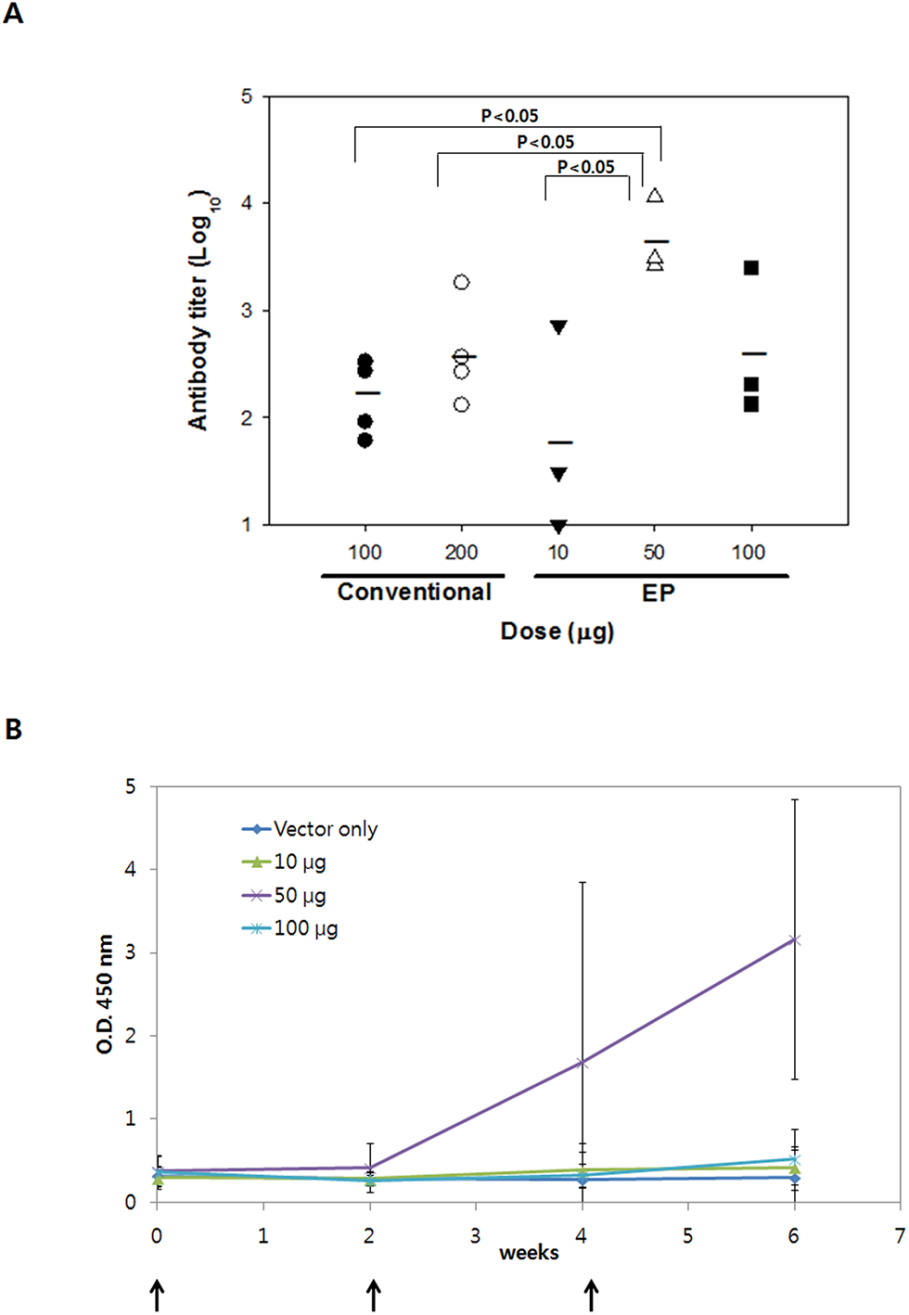
DNA dose effect on kinetics of PA-D4-specific antibody response elicited by EP or conventional injection. (A) Antibody levels are expressed as endpoint titers obtained using the ELISA assay. IgM-D4/SV40 DNA at the indicated doses was injected into mice at 0, 2 and 4 wk, and sera were collected at 8 wk. Symbols show individual values; the bars represent mean titer for each group. *P* values were determined using one-way ANOVA followed by Tukey multiple comparison tests. (B) Antibody response following various doses of IgM-D4/SV40 DNA vaccine administration via EP. Mice were immunized three times with plasmids as indicated. Antibody levels are expressed as OD_450_ values after the serum samples were diluted 100-fold.

To enhance antibody responses to IgM-D4/SV40, DNA dose effect on the level of anti-PA-D4 IgG production was assessed. As shown in Fig 4A, when a dose of 200 μg of DNA was administered by conventional needle injection, the antibody titer was higher than when 100 μg of DNA was administered by conventional needle injection. When 50 μg of DNA was delivered by electroporation (EP), antibody responses in mice were rapidly and consistently induced (Fig 4B) and remained significantly higher than the responses to 200 μg of DNA administered by conventional needle injection. More importantly, even the lowest DNA dose (10 μg) delivered by EP resulted in the induction of similar levels of anti-PA-D4 IgG titers compared with those elicited by a 10-fold higher dose of DNA administered by conventional needle injection. Thus, EP-based delivery dramatically enhanced the potency of the IgM-D4/SV40 DNA vaccine.

### Immune responses

**Fig 5:**
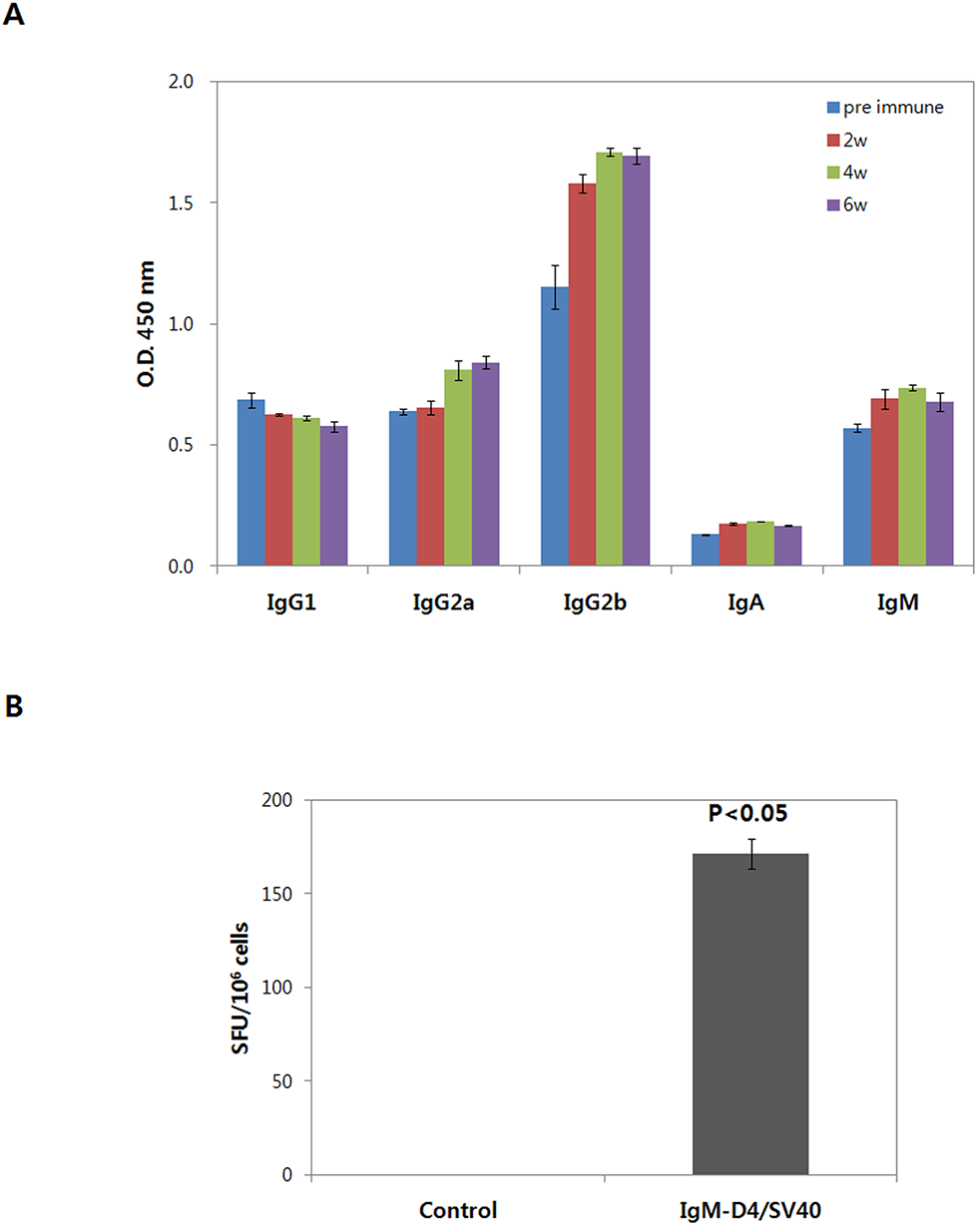
Immune response in mice immunized with IgM-D4/SV40 DNA vaccine. (A) Antibody analyses of IgG subclass after immunization with IgM-D4/SV40 DNA vaccine using EP delivery. The level of IgG subclass was measured by ELISA (1:100 dilutions). (B) DNA vaccination induces IFN-γ responses. Splenocytes from immunized mice were collected 8 weeks after first immunization, cultured in the presence of PA-D4 protein, and IFN-γ level measured by ELISPOT. Values are means ± standard deviations. *P*<0.05 compared to control.

In order to further characterize the immune response stimulated by IgM-D4/SV40, levels of the IgG subtypes were assessed. Mice had predominated production of the Th1-type with IgG2a and IgG2b subclasses, although low levels of IgG1 were also observed (Fig 5A). These results indicate that vaccination with IgM-D4/SV40 induces PA-D4-specific cell-mediated immune responses dominated by a Th1-type of reaction. In addition, increased IgA levels indicated that the IgM-D4/SV40 vaccine also induced the mucosal immune responses.

We next characterized the cell-mediated immune response that is generated with IgM-D4/SV40. IFN-γ enzyme-linked immunosorbent-spot (ELISPOT) assays were performed on splenocytes isolated from mice at 8 weeks after the initial injection. The presence of IFN-γ-producing cells suggests a Th1 type immune response. Cytokines were measured from spleen cells at 24 h after stimulation with recombinant PA-D4 protein. As shown in Fig 5B, while there was no increase in response from the mice immunized with pcDNA 3.1, IFN-γ production of splenocytes from mice immunized with IgM-D4/SV40 was significantly increased (*P*<0.05). These results suggest that the cellular immune response was also induced in mice vaccinated with IgM-D4/SV40.

### Biodistribution of plasmid IgM-D4/SV40 DNA vaccine

**Fig 6:**
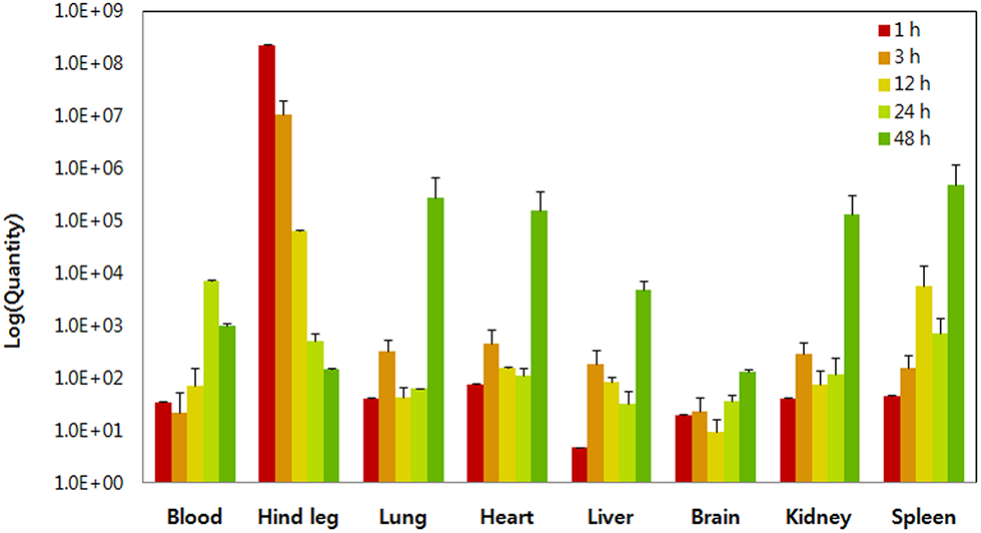
Biodistribution of DNA vaccines. Mice were immunized with 3.5 × 10^13^ copies IgM-D4/SV40 plasmid DNA and plasmid copy numbers were measured with quantitative real-time polymerase chain reaction assay at various points after IM injection. PCR reaction products were not detected for control animals (vector only). Data are expressed as PCN per microgram of DNA. Values are means ± standard deviations.

To analyze the distribution profiles of the IgM-D4/SV40 DNA vaccine, we used qPCR to quantify the level of DNA vaccine in various tissues. In a mouse time course biodistribution study, 3.5 × 10^13^ copies IgM-D4/SV40 plasmid DNA were administered intramuscularly and pharmacokinetic analysis of the plasmid DNA in the tissues was performed. The distribution of plasmid DNA in the tissues was measured from 1 h to 48 h after injection. The total DNA was extracted from the lung, liver, spleen, heart, brain, kidney, blood, and muscle at the different time points. Plasmid DNA copies were detectable in all tissues 48 h post-administration, although levels of plasmid DNA copies differed between tissues (Fig 6). One hour after injection of plasmid DNA, the highest copy numbers were observed in the injected muscles. However, 3 h post-administration, the copy numbers rapidly decreased. At 48 h after administration of plasmid DNA, plasmid copies remaining in the muscle decreased 1.5 × 10^6^-fold relative to the levels at 1 h. The level of plasmids in most of the organs increased substantially over time. The plasmid level in the spleen increased 10^5^-fold by 48 h post-administration compared with the early time point (1 h). The plasmid levels in organs were detected at 48 h in the following order: spleen, lung, heart, kidney, liver, and brain. However, the blood showed a different biodistribution profile in which the plasmid copies peaked at 24 h post-administration and rapidly declined thereafter.

### Immunization of guinea pigs

**Fig 7:**
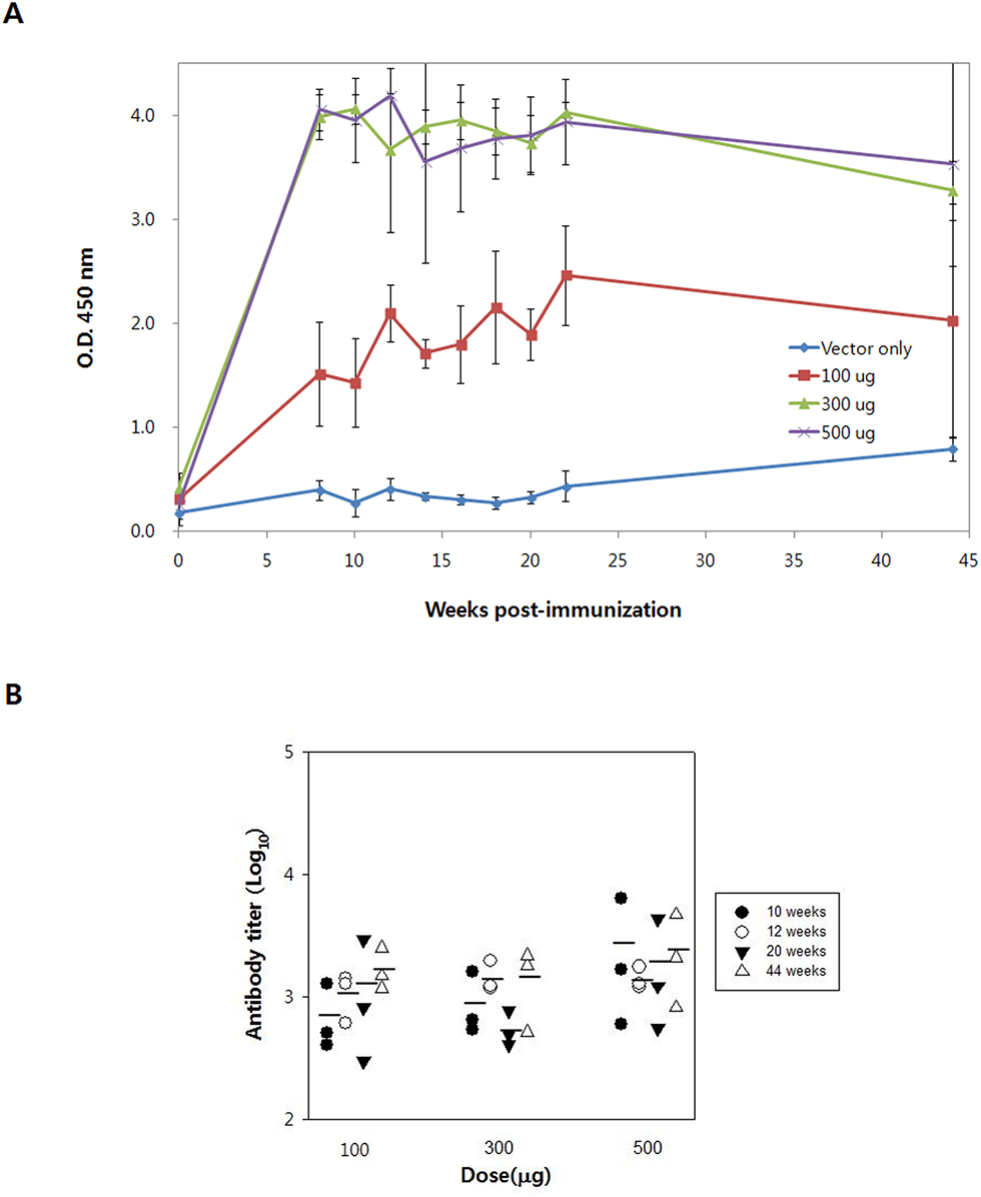
PA-D4-specific antibody responses in guinea pigs immunized with IgM-D4/SV40 DNA vaccine by EP. Guinea pigs were immunized 3 times (week 0, 3, and 6) with different doses of DNA vaccine. (A) Anti-PA-D4 IgG levels are expressed as OD_450_ values after the serum samples were diluted 100-fold. (B) Anti-PA-D4 IgG levels are expressed as endpoint titers obtained from the ELISA assay. Each symbol represents data from an individual guinea pig. The bars represent the mean titer for each group.

Next, to assess the effect of IgM-D4/SV40 DNA vaccine in a species of large body mass, guinea pigs were immunized with vaccine doses of 100, 300, and 500μg. Anti-PA-D4 IgG was measured for 44 weeks following three immunizations with EP. As shown in Fig 7A, administration by EP resulted in the rapid and consistent development of high titers of antibodies to PA-D4 for approximately 1 year. However, we noted no significance difference (*P*>0.05) in the anti-PA-D4 ELISA titers among the groups vaccinated with the 100, 300 and 500μg dose (Fig 7B).

### Protection against challenge with *B*. *anthracis*

**Fig 8:**
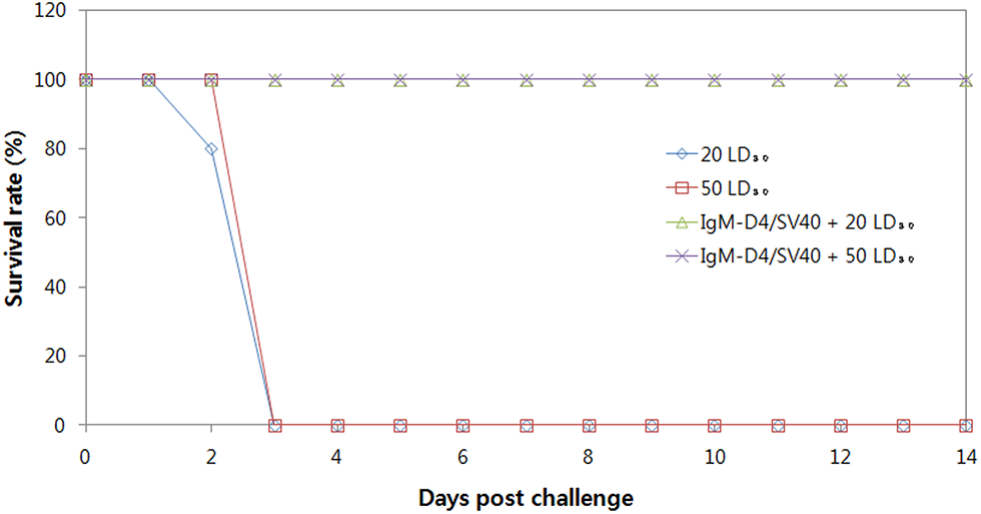
Survival of A/J mice immunized with IgM-D4/SV40 DNA vaccine. Mice were immunized with 50 μg of DNA vaccine intramuscularly via EP followed by two boosters with the same dose at 2-week intervals. A/J mice immunized with DNA vaccines were challenged by either 20 LD_50_ or 50 LD_50_ spores 2 weeks after the final immunization.

To determine if the immune response generated against PA-D4 was sufficient for providing protection against anthrax, we performed an *in vivo* protection experiment. A/J mice (n = 5∼6) were immunized IM via EP with 50μg of IgM-D4/SV40. The mice received two boosters with the same dose at a 2-week interval. Two weeks after the last immunization, mice were challenged with live spores. A/J mice received two different doses of *B*. *anthracis* Sterne spores subcutaneously, either 3.5 x 10^3^ (20 LD_50_) or 9 x 10^3^ (50 LD_50_). As expected, all mice that were immunized with control groups succumbed to challenge by day 3 and all mice that were immunized with IgM-D4/SV40 survived (Fig 8). In addition, survivors demonstrated high geometric titers (1.86 x 10^4^) of PA-D4-specific antibody one week before challenge (data not shown). From these results, we infer that the IgM-D4/SV40 DNA vaccine is able to generate protection against *B*. *anthracis*.

## Discussion

The protective immunity against infection with *B*. *anthracis* is entirely based on a response PA. Previous results suggest that the effectiveness of animal and human vaccines depends on the induction of anti-PA antibodies [11]. Thus, the induction of PA-specific antibodies is believed to be a key requirement for efficient anthrax vaccine development. The current licensed anthrax vaccine consists principally of PA adsorbed onto aluminum hydroxide (USA) and alum (UK). These vaccines are contaminated with trace amounts of LF and EF that cause transient side effects such as edema and local pain, and multiple injections are required for a base immunization [15].

Biowarfare and bioterrorism may require the vaccination of large populations against the *B*. *anthracis* infection. An ideal vaccine would be able to induce rapid immunity without causing adverse side effects. DNA vaccines fit most of these criteria. DNA vaccines can be produced quickly with high purity and low production cost. DNA vaccines consist of closed circular bacterial plasmids containing the bacterial origin of replication, a selectable marker such as an antibiotic resistance gene, and a gene insert expressing a vaccine immunogene or cytokine adjuvant under the control of a strong mammalian promoter and polyadenylation signal for good transcriptional expression in the vaccines [16,36]. Given these considerations, we developed and tested various plasmid candidates for anthrax DNA vaccines. The results from our study demonstrate that the eukaryotic expression plasmid containing the SV40 enhancer and signal sequence for secretion of encoded codon-optimized D4 of PA induces an antibody response against anthrax.

It has previously been demonstrated that DNA vaccination, using plasmid coding for PA83 or PA63, is able to protect mice against challenge with *B*. *anthracis* lethal toxins or spores [19,21]. Furthermore, DNA vaccination has been used to successfully protect nonhuman primates against anthrax [37]. However, concerns have been raised about administering the potentially active toxin subunits, PA83 or PA63, to humans at risk of being exposed to *B*. *anthracis*. Because it is unknown how toxins made during *B*. *anthracis* infection will interact with PA produced from a genetic vaccination, we chose to use only D4 of PA as a vaccine antigen. PA-D4, which is a host cell receptor-binding region, is not functional as a toxin subunit since it lacks the domains necessary for both oligomerization and interaction with other toxin components, LF and EF [1,3]. Our results demonstrate that PA-D4 successfully elicited antibodies to protect against the antigen.

The dramatic differences in codon usage between bacteria and mammals may have an effect on the expression of *B*. *anthracis* genes in transfected cells in the mammalian host. Each amino acid can be encoded by several different synonymous codons. The frequencies at which these synonymous codons are used depend on the level of protein expression and differ among organisms. In general, highly expressed genes are biased toward codons that are recognized by the most abundant tRNA species in the organism [28,29]. We observed that the difference between the original PA-D4 and codon-optimized PA-D4 codon usage was 30% (data not shown). In our eukaryotic expression system, plasmids containing the codon-optimized PA-D4 gene showed successful protein expression in mammalian cells. Human codon-optimized PA83 expression plasmid is an accepted model for testing immunogenicity and protection induced by candidate anthrax vaccines [19]. Codon optimization of PA-D4 may improve expression of the *B*. *anthracis* gene in 293T cells by a number of possible mechanisms. One of many possibilities is that the use of common mammalian codons may improve the rate of translation by avoiding the requirement for tRNA to be present at stable but low concentrations [29]. By contrast, gene expression of original PA-D4 was not detected in our studies. It was previously reported that expression of PA-D4 failed even with codon optimization [38]. A clear understanding of the mechanism of improved expression would allow for a more rational design of optimized anthrax DNA vaccines in the future.

Secreted antigens are likely endocytosed and then enter the exogenous pathway of antigen representation by the MHC class II pathway. There have been various DNA vaccination studies, indicating that secreted antigen induces better immune responses than antigen expressed in the cytosol [17]. In the present study, we evaluated the potential effect of including a signal sequence in an expression vector intended for immunization as a strategy to enhance immunity responses to the encoded PA-D4 antigen. To investigate this, two DNA plasmids encoding the PA-D4 antigen were constructed. One of the plasmids, IgM-D4, had the IgM signal sequence preceding the encoding sequence. In comparison, the other plasmid, IgK-D4, carries the IgK signal sequence for secretion of PA-D4 antigen. Upon immunization, the translated signal peptide should direct the nascent PA-D4 protein into the ER and Golgi pathway of the transfected host cells, thus facilitating efficient secretion of the PA-D4. An IgM leader sequence directed efficient secretion of eukaryotic protein in the baculovirus system [31]. Inclusion of DNA sequences encoding the secretory signal sequence of IgK light chain into DNA expressing glycoprotein E2 antigen of BVDV was employed for vaccine purpose [32]. The expression of signal peptide bearing PA-D4 was characterized *in vitro* by transfection of 293T cells. It was clearly demonstrated that both versions of PA-D4 were expressed at different levels. PA-D4 protein was found in culture media, suggesting that PA-D4 was targeted in the secretory pathway.

The immune response of a DNA vaccine may be enhanced by the high-level of transgene expression. SV40 enhancer has been shown to enhance gene expression in various cell types and facilitate nuclear transport of plasmids from the cytoplasm *in vitro* [34]. Although it has been previously reported that inclusion of the SV40 enhancer in the plasmid vectors increased the gene expression level of the transgene *in vivo* by as much as 20-fold [35], little is known about whether the immune response is enhanced. Our results showed that anti-PA-D4 IgG titers obtained from mice injected with plasmids carrying a 72-bp tandem repeat from an SV40 enhancer are approximately 10-fold higher than those from mice injected with plasmids without the SV40. We also observed long-term effects of DNA vaccines carrying SV40, which showed stable enhanced anti-PA-D4 IgG production levels for approximately 1 year. Therefore, our results clearly showed that the strategy of inserting a SV40 enhancer upstream of the CMV promoter in the backbone of the plasmid may be a very important tool for improving the immunogenicity of the DNA vaccine.

The effectiveness of our IgM-D4/SV40 DNA vaccine in inducing Th1 and Th2 responses was studied. In general, DNA vaccines are considered strong inducers of Th1 responses. In the current study, we showed a less balanced immune response of both Th1 and Th2 types, and it was observed that Th1 skewed the reaction, which suggests that the DNA vaccination elicited a strong cell-mediated immune response. This was further confirmed by our data regarding IFN-γ activation obtained from ELISPOT assays using restimulated spleen cells from mice vaccinated with IgM-D4/SV40. In addition, the level of Th2 antibody isotype IgG1 also gradually increased in DNA-vaccinated mice, suggesting that IgM-D4/SV40 elicited both humoral and cell-mediated immunity. Clearly, induction of a Th1/Th2 mediated immune profile is a desirable advantage for protection against anthrax. Previous reports have shown that both Th1 and Th2 responses were induced following immunization with a DNA plasmid encoding PA [18].

One of the issues regarding DNA vaccine efficiency is the mode of its delivery. Conventional injection of DNA is considered a key limitation due to inefficient uptake by cells. In this regard, electroporation appears to be a promising approach for improving the immunogenicity of DNA vaccines because increasing cellular permeability results in a high level of protein expression and improved immune response. Recently, clinical trials have confirmed the efficiency of EP [39]. To develop an effective vaccine against anthrax, we choose a short vaccination schedule in which animals were primed with DNA at time zero, boosted with DNA 2 times at 2-week intervals. The repeated vaccination with PA-D4, which contains the dominant protective epitopes of PA, may result in the induction and maintenance of high frequencies of Th1/Th2 immune cells. In our results, EP greatly improved dose efficiency of the IgM-D4/SV40 vaccine, inducing a high level of immune response even at a low dose of 10 μg. In addition, the EP-base vaccination exhibited more rapid production of anti-PA-D4 IgG than the conventional needle injection, which did induce an antibody response as early as 3 weeks after the first vaccine immunization. Previous work suggests that characteristics of EP, such as low dose of DNA requirement and fast onset of immune response, are of interest for the development of vaccines for biodefense [40]. Furthermore, a strong immune response was also achieved when the IgM-D4/SV40 vaccine was delivered by EP in guinea pigs (a large mammalian animal model). In addition, a body weight-monitoring study with guinea pigs showed no toxicity (data not shown), suggesting that the IgM-D4/SV40 DNA vaccine was safe, which would be a pre-requirement of any clinical application. In line with our results, previous studies have shown the ability to stimulate a humoral response and confer protection in a guinea pig model of anthrax following PA carrying plasmid immunization [41].

The purpose of the biodistribution study was to investigate the target tissue and clearance profile of the DNA vaccine. In the present study, the biodistribution of the IgM-D4/SV40 DNA vaccine was analyzed with a validated qPCR method, which is one of the most sensitive nucleic acid detection techniques to assess DNA levels in tissues [25,26]. From the results of our qPCR assay study, plenty of plasmid DNA was detected at the injection site soon after IM injection. However, plasmid DNA at the injection site decreased rapidly, probably due to transfer or degradation. When low levels of plasmid DNA were detected at the injection site, high levels were present predominately in various organs such as the spleen, lung, kidney, heart, and liver. This may indicate that translocation of significant plasmid DNA from the injection site to other tissues was through the circulation of blood. Taken together, a significant amount of IgM-D4/SV40 DNA vaccine was detected in all of the tissues examined. These results were in accordance with previous reports such as those of Zhang et al [42] and Liu et al [43]. In their research, a majority of IM-injected plasmid DNA rapidly spread from the injection site to various organs. In particular, the highest level of IgM-D4/SV40 DNA vaccine occurred in the spleen, suggesting the production of an effective immune response. Previous reports suggest that the long-term existence of plasmid DNA in lymphatic organs implies the correlation of distribution of plasmid DNA and the induction of an effective immune response [42,44].

Finally, it was reported that the 50% lethal dose for A/J mice injected subcutaneously with *B*. *anthracis* Sterne spores is 1.1 x 10^3^ [45]. However, there appear to be differences in virulence between different methods of spore preparation. The determined LD_50_ in our study was 1.7 x 10^2^ spores, which is approximately 6 times lower than the previously reported value. A/J mice have been found to be much more susceptible to Sterne infection than other strains of mice, such as BALB/c mice [4]. We showed that a high level of protection against *B*. *anthracis* of up to 50 LD_50_ was afforded by vaccination with IgM-D4/SV40. This reflects the robust immune response elicited by IgM-D4/SV40. In conclusion, our results demonstrate that the IgM-D4/SV40 vaccine delivered by EP is able to induce a rapid, robust, and durable protective immune response with a low DNA dose. These findings suggest that the IgM-D4/SV40 vaccine may represent a promising next-generation anthrax vaccine candidate. However, further work is needed to optimize our results for clinical application
